# Advancing Alzheimer's Diagnosis: Creating the Gold Standard pT217‐Tau Antibody for Enhanced Sensitivity

**DOI:** 10.1002/alz.088529

**Published:** 2025-01-09

**Authors:** Ji‐Seon Park, Heeseon Park, Mi‐Hyang Cho, Kyung‐Hoon Hwang, Min‐Seok Kim

**Affiliations:** ^1^ ADEL Institute of Science & Technology (AIST), Seoul Korea, Republic of (South); ^2^ ADEL Institute of Science & Technology (AIST), ADEL, Inc., Seoul Korea, Republic of (South)

## Abstract

**Background:**

While numerous blood biomarkers have been proposed for Alzheimer’s disease (AD), only a few have demonstrated definitive diagnostic value. Recently, a set of phosphorylated Tau proteins, particularly pT217, have emerged as promising candidates with superior diagnostic performance. Given the development of pT217 antibodies by major global pharmaceutical companies, our goal is to create the best‐in‐class pT217 antibody, establishing it as the gold standard for diagnostics. To address common practical challenges encountered in applying antibodies to patient blood samples, including plasma or serum, we implemented specific strategies from the initial screening of antibodies targeting pT217. We then compared the performance of our various antibody clones against those of other companies.

**Methods:**

To develop antibody specifically targeting pT217 Tau, we obtained antibody clones from mice immunized with an antigen including epitope targeting pT217 sequence. Using several Tau fragment peptides containing pT217 sequence, we compared the binding efficiency of the pT217 antibody clones by ELISA analysis. Additionally, we explored diagnostic potential of the clones for AD in human plasma samples.

**Results:**

Our antibody clones exhibited a threefold enhancement in the lower limit of quantification (LLOQ) for pT217 Tau fragments compared to antibodies from Company‐L. Additionally, the LLOQ for different lengths of pT217 Tau fragments was improved fourfold with our antibody clones when contrasted with Company‐L's antibodies. These results unequivocally underscore the significantly higher sensitivity of our antibody candidates compared to those from Company‐L.

**Conclusions:**

Our study has demonstrated the successful development of pT217 antibodies with superior sensitivity in comparison to global competitors' pT217 antibodies. These antibodies exhibit robust and specific binding to the pT217 Tau peptide, a pivotal component in Alzheimer's disease diagnosis and research. The heightened sensitivity of our antibodies positions them as strong contenders in advancing Alzheimer's disease diagnostics, potentially enabling earlier and more accurate disease detection. These findings hold substantial promise for enhancing early Alzheimer's disease diagnosis and contributing to the development of more effective treatment strategies.

